# Intraneural Lipoma of the Digital Nerve: A Case Report and Literature Review

**DOI:** 10.7759/cureus.13074

**Published:** 2021-02-02

**Authors:** Jeffrey DeSano, Preston Gardner, Justin W Hart, Samson Samuel, Keoni Williams

**Affiliations:** 1 Plastic and Reconstructive Surgery, Beaumont Health, Royal Oak, USA; 2 Hand Surgery, Michigan Surgery Specialists, Warren, USA

**Keywords:** intraneural, lipoma, ulnar, digital, nerve

## Abstract

Intraneural lipomas are rare soft-tissue tumors that can occur particularly within the median nerve. Even fewer cases have been reported of their occurrence within the ulnar nerve. These masses can cause compression neuropathies. In this report, we present the first documented case of an intraneural lipoma of an ulnar digital nerve.

## Introduction

Intraneural lipomas and lipofibroma (librofibromatous harmatomas) are rare soft-tissue masses of peripheral nerves. These masses are benign adipose tumors that most commonly occur in the forearm and wrist. The first case of lipofibroma was reported in 1953 by Mason [1]. The first intraneural lipoma was reported in 1964 by Morley [2]. These masses may cause progressive compression neuropathies if they are large enough. Yet, there are significant differences between intraneural lipomas and lipofibromas [3]. Intraneural lipomas are usually well-demarcated, encapsulated masses that displace rather than infiltrate the nerves. Lipofibromas are infiltrative in nature, composed of fatty and fibrous tissue. Given their distinctness, these lesions are managed differently.

Intraneural lipomas may be amenable to complete excision without sacrificing the offending peripheral nerve. Intraneural lipomas have a female predominance and, although rare, most commonly occur within the median nerve in the forearm and wrist [4]. Some cases have been reported within the ulnar nerve and radial nerve [5-8]. All reported upper extremity cases include the forearm proximally to the carpal tunnel and Guyon’s canal distally. Nothing further distal has been reported in the literature to date. Due to this scarcity, we present here the first documented case of an intraneural lipoma of an ulnar digital nerve, which extended to the level of the distal interphalangeal joint.

## Case presentation

**Figure 1:**
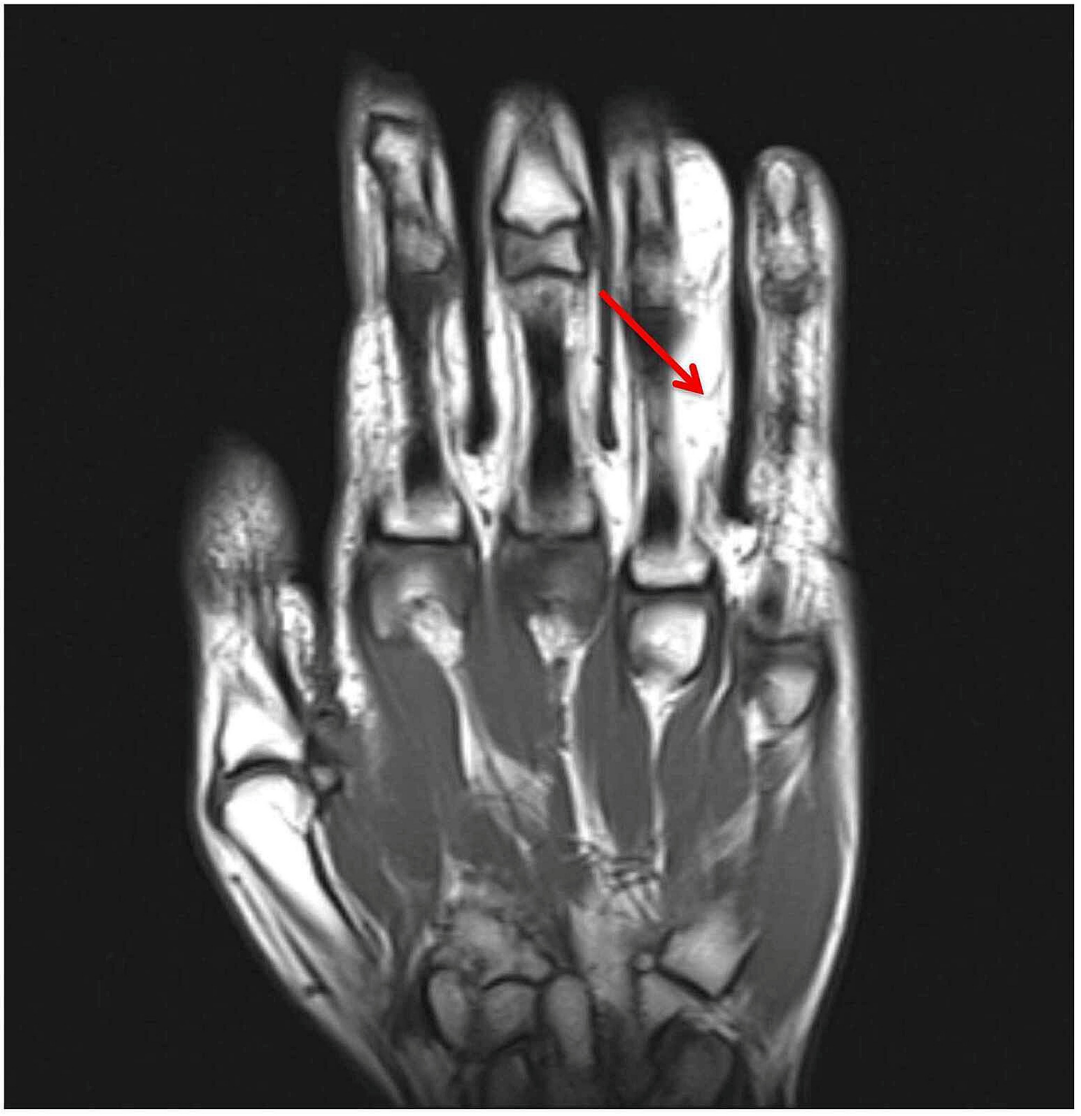
MRI image of the right hand without gadolinium contrast enhancement. The mass demonstrated a hyperintense signal on coronal T1-weighted imaging (arrow) MRI: magnetic resonance imaging

**Figure 2:**
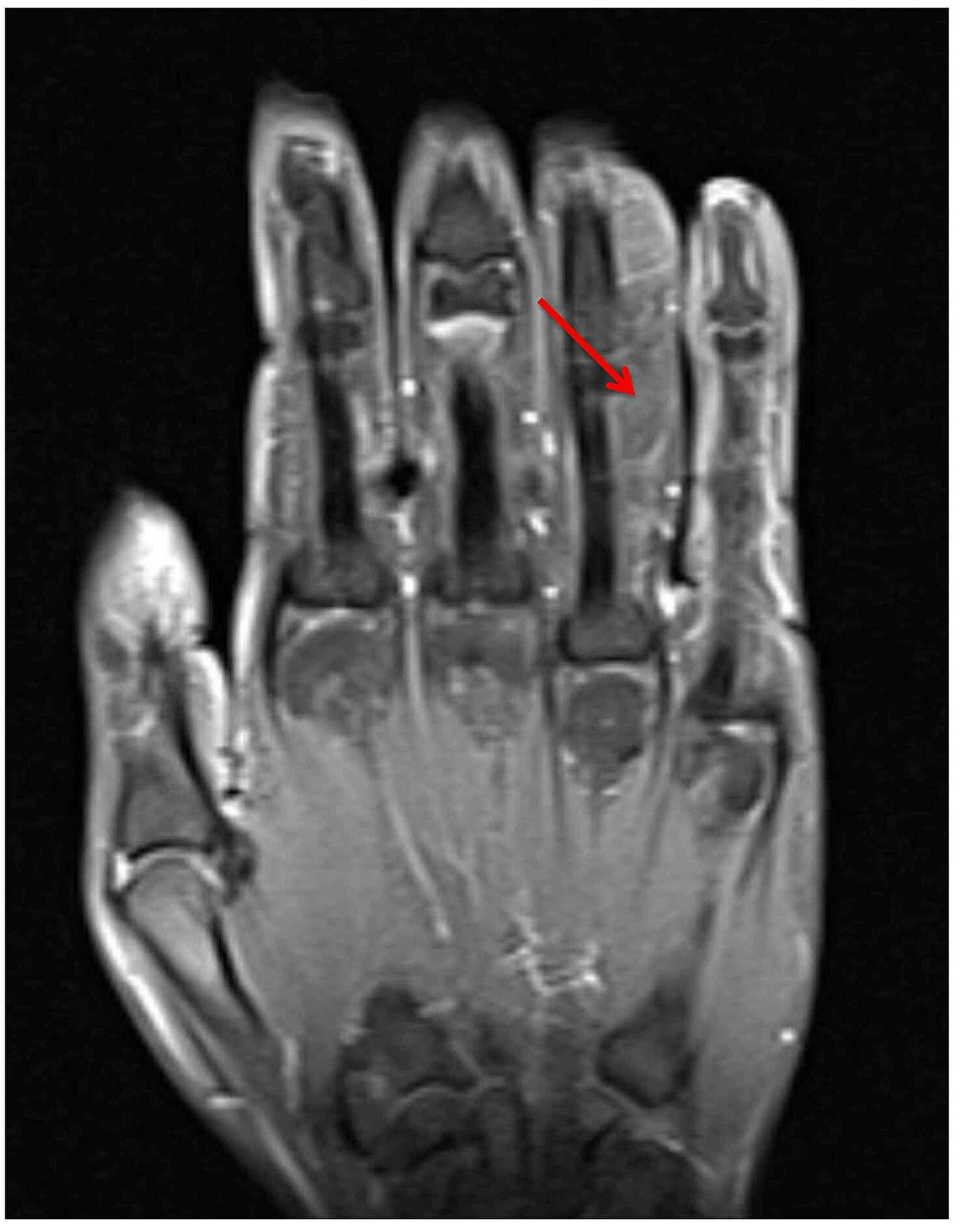
MRI image of the right hand without gadolinium contrast enhancement. The mass demonstrated complete fat saturation on coronal T2-weighted imaging (arrow) MRI: magnetic resonance imaging

**Figure 3:**
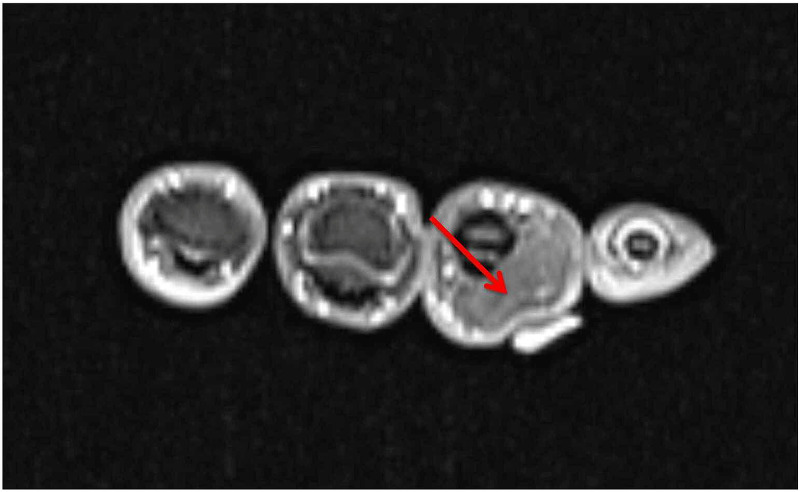
Axial MRI imaging of the right hand without gadolinium contrast enhancement; mass noted (arrow) MRI: magnetic resonance imaging

**Figure 4:**
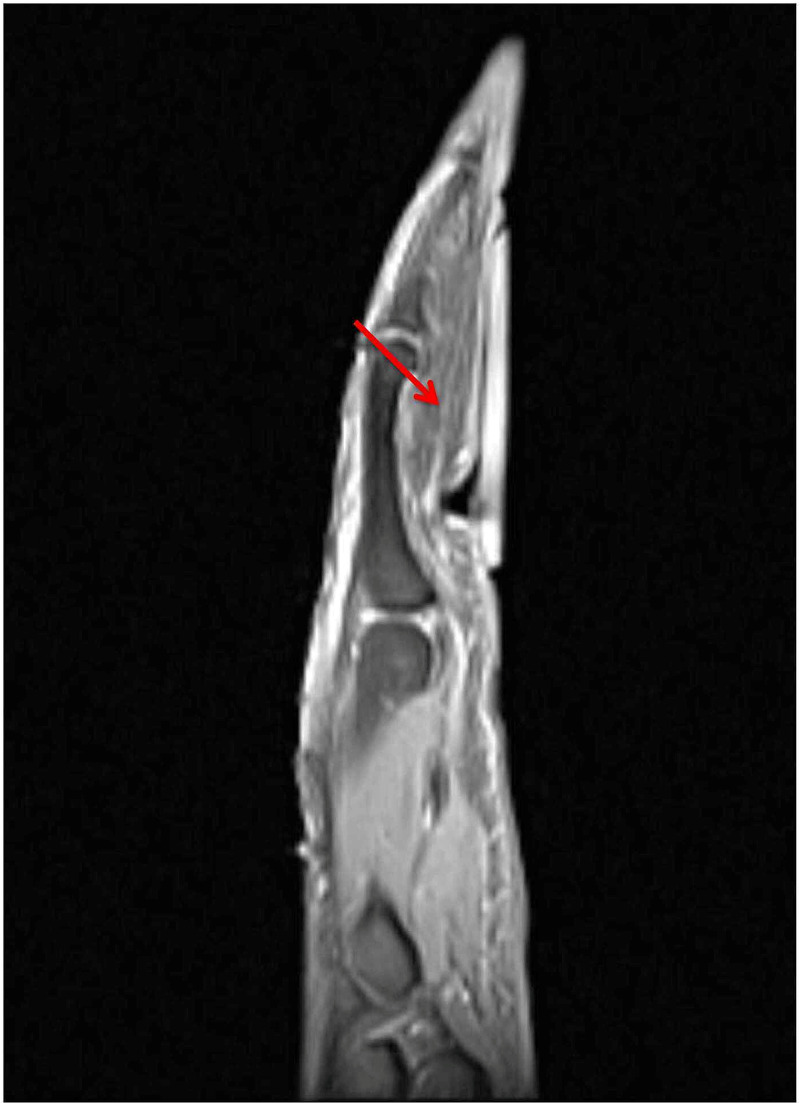
Sagittal MRI imaging of the right hand without gadolinium contrast enhancement showed intraneural lipoma extending along volar right ring finger MRI: magnetic resonance imaging

A 25-year-old, right-hand-dominant female presented with a progressively enlarging mass along the ulnar border of her right ring finger. The mass extended proximally from her proximal phalanx to her middle phalanx, ~2 x 2 cm in size. The mass was soft, mobile, with no overlying skin changes. No pain, tenderness, paresthesias, or bruit were noted. There was no history of trauma. MRI was obtained and demonstrated a lobulated lipoma on the volar and ulnar aspect of the right ring finger extending from the proximal phalanx to the distal aspect of the middle phalanx (Figure 1, Figure 2, Figure 3, Figure 4).

Surgical excision was recommended, which the patient deferred at that time. The mass continued to enlarge over the next two years with the onset of worsening pain and paresthesias. Repeat MRI confirmed the diagnosis of intraneural lipoma of the ulnar digital nerve of the patient’s right ring finger. The patient agreed to surgical intervention.

**Figure 5:**
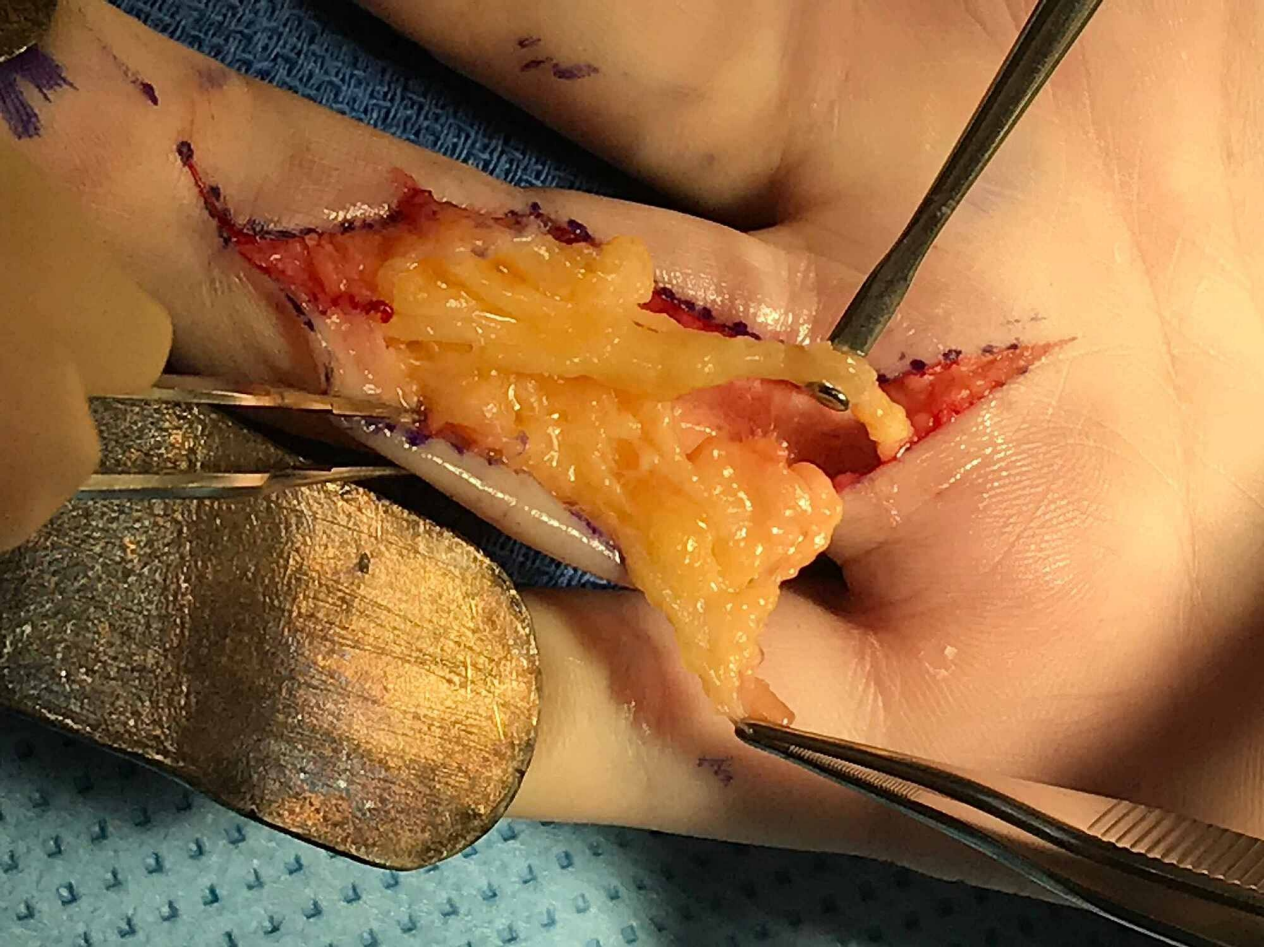
Intra-operative photo showing a large, multilobulated, fibrofatty tumor consistent with an intraneural lipoma intimately associated with the ulnar digital nerve The lipoma extended from the level of the A1 pulley proximally to the level of the distal interphalangeal joint distally

Upon exploration, the mass appeared to be a large, multilobulated, serpiginous, fibrofatty tumor consistent with an intraneural lipoma along the entire volar ulnar surface of the ring finger (Figure 5).

**Figure 6:**
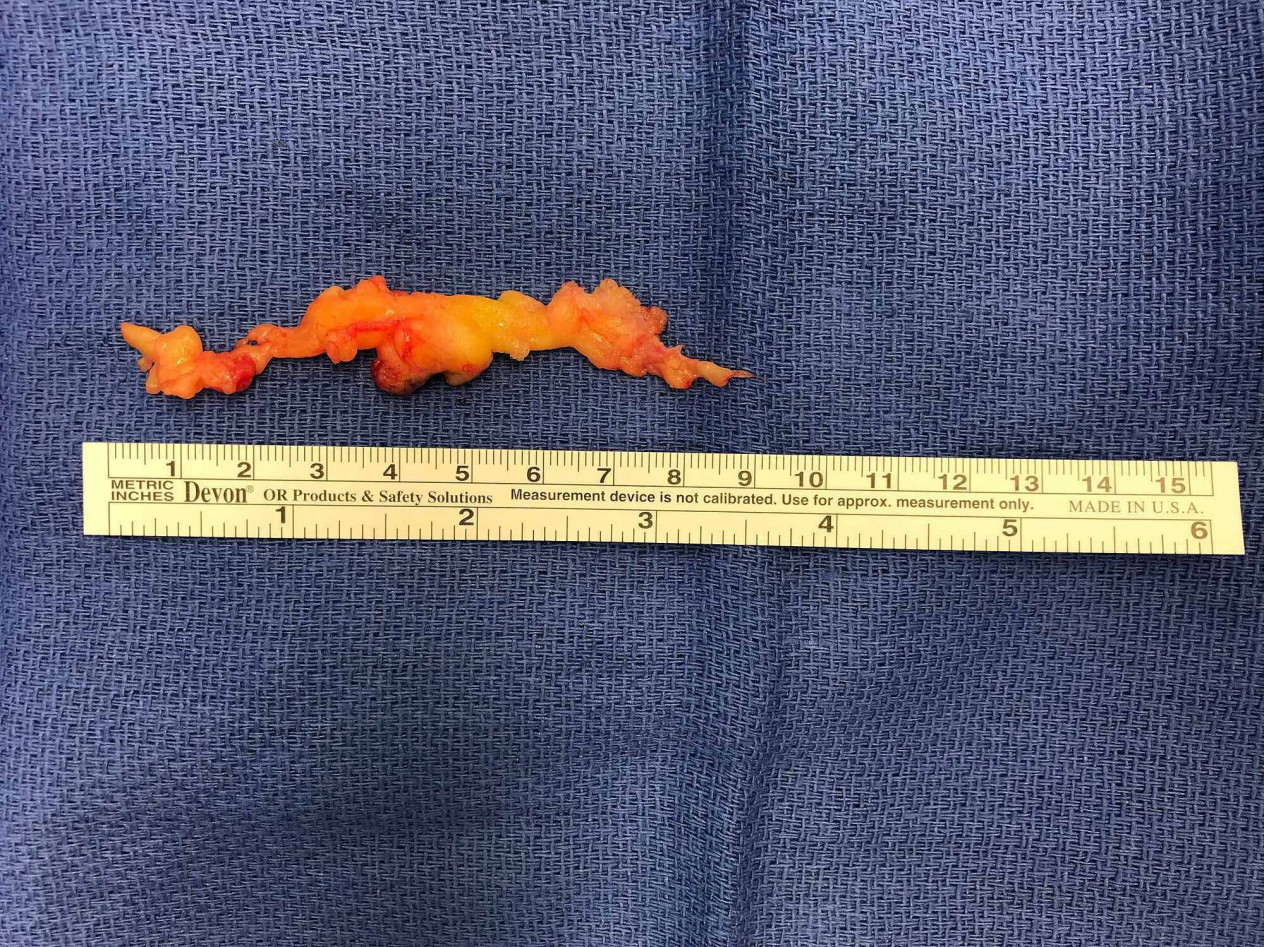
The resection yielded a 9-cm multilobulated, serpiginous, fibrofatty mass

The lipoma was intimately associated and intertwined with the ulnar digital nerve, extending from the level of the A1 pulley proximally to the level of the distal interphalangeal joint distally. The ulnar digital artery was displaced by the mass. Meticulous dissection was undertaken to enucleate the intraneural lipoma, safeguarding against any injury to the ulnar digital nerve. The resection yielded a 9-cm multilobulated, serpiginous mass with no residual lesion (Figure 6).

**Figure 7:**
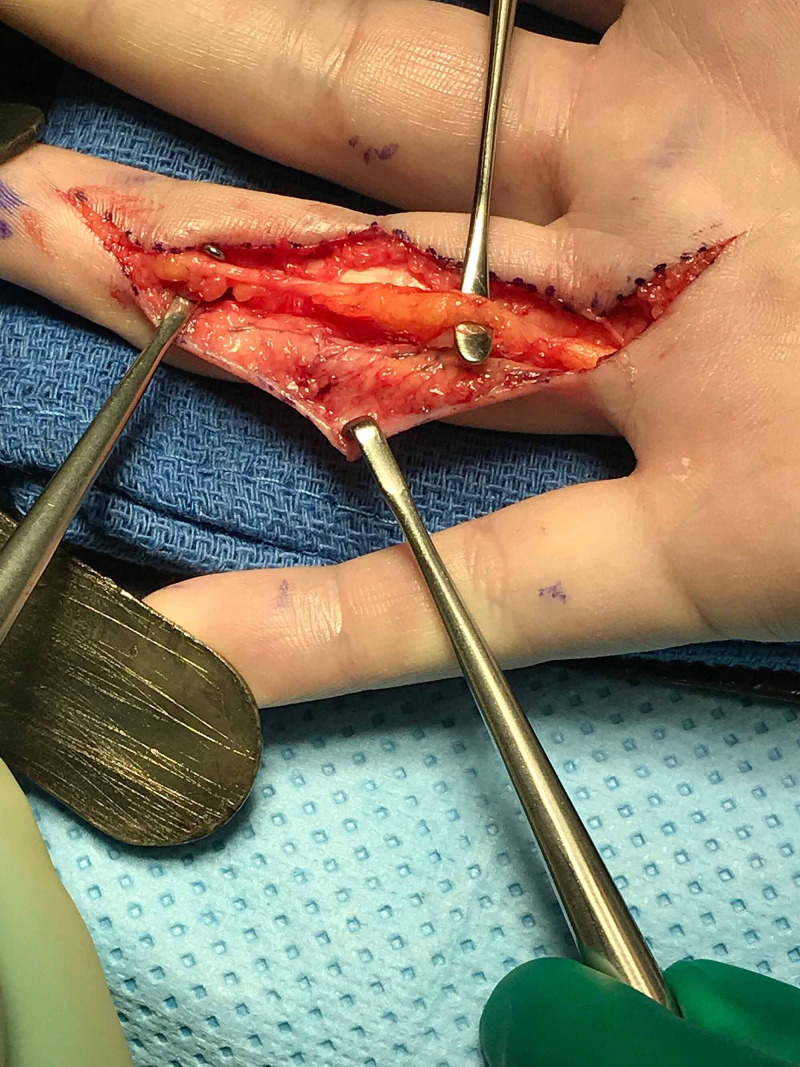
After meticulous dissection to completely excise the intraneural lipoma, the ulnar digital nerve remained intact with noted areas of the previous compression

After meticulous dissection to completely excise the intraneural lipoma, the ulnar digital nerve remained intact with noted areas of the previous compression (Figure 7).

Gross pathological examination of the specimen revealed a large, encapsulated mass comprised of fibroadipose tissue consistent with an intraneural lipoma. Postoperatively, the patient experienced some mild paresthesias along the ulnar aspect of her right ring finger, which did resolve over time. No strength or range-of-motion deficits were observed. Full recovery was achieved by the patient.

## Discussion

Adipose tumors are the most common soft-tissue tumor; yet, in the hand, lipomas account for less than 5% of benign tumors [9,10]. Additionally, lipomas of peripheral nerves are exceedingly rare. If large enough, these masses can extrinsically cause symptomatic compression neuropathies. Their natural history is slow-growing, but progressive enlargement could result in pain, paresthesias, and decreased motor function.

Intraneural lipomas and lipofibroma (librofibromatous harmatomas) are rare benign adipose tumors of peripheral nerves that most commonly occur in the forearm and wrist. The first lipofibroma was reported in 1953 by Mason [1]. The first intraneural lipoma was reported in 1964 by Morley [2]. Clinically, these masses can present in a similar manner. Yet, there are significant differences between intraneural lipomas and lipofibromas [3]. Intraneural lipomas are usually well-demarcated, encapsulated masses that displace rather than invade the nerves. Lipofibromas, on the other hand, are diffusely infiltrative, composed of fatty and fibrous tissue. Given their pathological differences, these lesions are managed differently.

MRI is diagnostic and helpful in distinguishing between the two entities [11]. This imaging is crucial to aid in treatment planning and patient counseling. Intraneural lipomas are encapsulated and noninvasive, which makes them amenable to complete excision without nerve sacrifice. They cause compression neuropathies due to mass effect and displacement. Lipofibromas are invasive and fibrotic in nature, invading and enveloping neural elements and fascicles. Usually, complete excision of lipofibromas cannot be achieved without extensive interfascicular resection if not complete nerve sacrifice. Surgical intervention of lipofibromas then may include mass enucleation, complete nerve resection with subsequent nerve grafting and/or transfer. Since these surgical methods are vastly different from each other, MRI should be obtained to adequately advise the patients and aid surgical planning.

Intraneural lipomas, although rare, most commonly occur within the median nerve in the forearm and wrist [4]. Some cases have been reported within the ulnar nerve and radial nerve [5-8]. All reported upper extremity cases include the forearm proximally to the carpal tunnel and Guyon’s canal distally. To our knowledge, no cases of an intraneural lipoma of a digital nerve have been reported. Due to this scarcity, we presented here the first documented case of an intraneural lipoma of an ulnar digital nerve, which extended to the level of the distal interphalangeal joint. Complete excision of the intraneural lipoma was planned and discussed with the patient. Meticulous dissection achieved complete removal of the intraneural lipoma, without injury to the ulnar digital nerve and artery. The ulnar digital nerve remained intact with noted areas of mass-induced compression. Postoperatively, some neurapraxia was noted clinically. These symptoms did resolve over a short period of time. Thus, complete excision of the intraneural lipoma of the ulnar digital nerve and full recovery was achieved.

## Conclusions

In this report, we presented the first documented case of an intraneural lipoma of an ulnar digital nerve and its successful complete excision. Benign fibroadipose tumors of the upper extremity are extremely rare. Progressive compression neuropathy symptoms may be noted given the tumor’s location and size. Surgical excision should be considered in patients with large masses, especially if symptomatic. MRI can help differentiate between an encapsulated, intraneural lipoma, which is amenable to complete excision, and an infiltrating lipofibroma, which may only be completely removed with nerve resection/sacrifice.
